# Both IFN-γ and IL-4 Induce MHC Class II Expression at the Surface of Mouse Pro-B Cells

**DOI:** 10.1155/1997/76506

**Published:** 1997

**Authors:** Suzanne Lombard-Platet, Valerie Meyer, Rhodri Ceredig

**Affiliations:** 1 Laboratoire de Génétique Moléculaire des Eucaryotes du CNRS et Unité 184 de ľ INSERM Institut de Chimie Biologique 11 rue Humann Strasbourg 67085 France; 2 Basel Institute for Immunology 487 Grenzacherstrasse Basel CH-4005 Switzerland

**Keywords:** MHC class II, lnvariant chain, IL-4, IFN-γ, pro-B cells

## Abstract

Pro-B cells are early B-cell progenitors that retain macrophage potential. We have studied MHC class II molecules and invariant chain inducibility on four class II negative mouse pro- B-cell clones. We analyzed the effects of IL-4 and IFN-γ, which represent the major inducers of class II in the B-lymphoid and monocytic/macrophage lineages, respectively. After 48 h of treatment with either cytokine, three pro-B-cell clones (C2.13, A1.5, and F2.2) expressed intracellular invariant chain and cell-surface class II molecules. One clone (D2.1) remained negative. As already reported, more differentiated 70Z/3 pre-B cells were inducible by IL-4 only. These data suggest that the induction of class II and invariant-chain genes are subject to regulation throughout B-cell differentiation.


*Developmental Immunology*, 1997, Vol. 5, pp. 115–120
Reprints available directly from the publisher
Photocopying permitted by license only

© 1997 OPA (Overseas Publishers Association)
Amsterdam B.V. Published under license in The Netherlands
under the Harwood Academic Publishers imprint
part of The Gordon and Breach Publishing Group
Printed in Malaysia

*Short Communication*

### Both IFN-γ and IL-4 Induce MHC Class II Expression at the Surface of Mouse Pro-B Cells

SUZANNE LOMBARD-PLATET[a], VALERIE MEYER[a] and RHODRI CEREDIG[b,*]

[a]*Laboratoire de Génétique Moléculaire des Eucaryotes du CNRS et Unité 184 de l'INSERM, Institut de Chimie Biologique, 11 rue Humann, 67085 Strasbourg, France;* [b]*Basel Institute for Immunology, 487 Grenzacherstrasse, CH-4005, Basel, Switzerland*

*(Received 4 December 1995; In final form 20 November 1996)*

Pro-B cells are early B-cell progenitors that retain macrophage potential. We have studied MHC class II molecules and invariant chain inducibility on four class II negative mouse pro-B-cell clones. We analyzed the effects of IL-4 and IFN-γ, which represent the major inducers of class II in the B-lymphoid and monocytic/macrophage lineages, respectively. After 48 h of treatment with either cytokine, three pro-B-cell clones (C2.13, A1.5, and F2.2) expressed intracellular invariant chain and cell-surface class II molecules. One clone (D2.1) remained negative. As already reported, more differentiated 70Z/3 pre-B cells were inducible by IL-4 only. These data suggest that the induction of class II and invariant-chain genes are subject to regulation throughout B-cell differentiation.

*Keywords:* MHC class II, Invariant chain, IL-4, IFN-γ, pro-B cells

### INTRODUCTION

The expression of MHC class II molecules is restricted to some cell types, including B cells, macrophages, dendritic cells, and thymic epithelium. Class II molecules are associated intracellularly with a monomorphic glycoprotein: the invariant chain (Ii). MHC class II and Ii genes are encoded on different chromosomes; coordinate expression is not absolute for a number of cases (Momburg et al., 1986; Viville et al., 1993). The promoter and enhancer regions of class II and Ii genes contain both shared and distinct control elements (Zhu and Jones, 1990). The level of class II surface expression can be modulated by different substances, cytokines, or antigenic stimuli. In both pre-B cells and mature B cells, class II molecules and the Ii chain can be induced, or up-regulated, by IL-4. IL-4 is also able to induce significant amounts of MHC class II cell-surface expression on peritoneal and bone-marrow-derived macrophages (reviewed in Glimcher and Kara, 1992). In macrophages and cells of the nonhematopoietic lineage, IFN-γ represents the major inducer. In contrast, IFN-γ can downregulate IL-4-mediated induction of class II molecules in B cells (Mond et al., 1986).

*Corresponding author.

The different stages of differentiation of early B-cell precursors to mature B cells have been described, and several nomenclatures have been proposed (reviewed by Hardy et al. 1994; and Rolink et al., 1994). During differentiation in the bone marrow, B-cell progenitors sequentially lose and/or acquire various antigens, and gradually start to rearrange their Ig locus. The stage at which MHC class molecules are first detectable has been recently debated. Pre-B cells have been shown to express significant amounts of class II molecules (Miki et al., 1992; Tarlinton, 1993; Hayakawa et al., 1994). We have recently generated IL-7 transgenic mice in which lymphoid tumors developed. These tumors are mostly comprised of pro-B and pre-B cells (Fisher et al., 1995, Mertsching et al., 1995). Cells harvested from the tumors have been cloned *in vitro* on a monolayer of bone-marrow stromal cells B16.14. Five pro-B-cell clones, originating from different tumors, have been derived. They express the following antigens: B220, BP-1, HSA, AA4.1, CD43, and have their IgH locus in germline configuration (Fisher et al., 1995). One of them, G12, spontaneously expresses at its cell surface small amounts of class II molecules (Lombard-Platet et al., 1995). This expression can be enhanced when the cells have been grown in the presence of IL-4. The other pro-B-cell clones do not spontaneously express either cell-surface or internal class II molecules nor do they express a detectable Ii chain. It has been shown that early B-cell progenitors retain a bipotential property for the generation of both B cells and macrophages (Cumano et al., 1992; Fisher et al., 1995). Because the control by cytokines, in particular IL-4 and IFN-γ, of MHC class II expression by mature B cells and macrophage differ, we decided to investigate the induction of class II expression by these cytokines in our pro-B-cell clones.

### RESULTS AND DISCUSSION

The *in vitro* growth of our pro-B-cell clones is dependent on cell contact and adhesion with the B16.14 stromal-cell line. Whereas the pro-B cells were loosely attached, B16.14 cells were strongly adherent to the tissue-culture plates. Pro-B cells were harvested by gentle pipetting; however, a few B16.14 cells could also be detached. These residual B16.14 cells were easily excluded for FACScan studies because they have different side-scatter (SSC) signals from the pro-B cells and were thus excluded from the analysis gate (not shown). Figures 1A and 1B show MHC class II staining of the C2.13 pro-B cells without induction, or after 48 hr of culture with saturating concentrations of IL-4 or IFN-γ. IL-4 treatment resulted in a strong induction of both invariant chain (Ii) and class II molecules. The latter were strongly expressed at the cell surface. Surprisingly, IFN-γ had the same, although weaker, effect. This induction was specific for class II molecules, because no variation was observed with other antigens such as LFA-1 (not shown). It must be noted that viability was affected by IFN-γ treatment. This cytokine is known to cause apoptosis of mouse pre-B cells after 3 days of culture (Grawunder et al., 1993). In our experi-

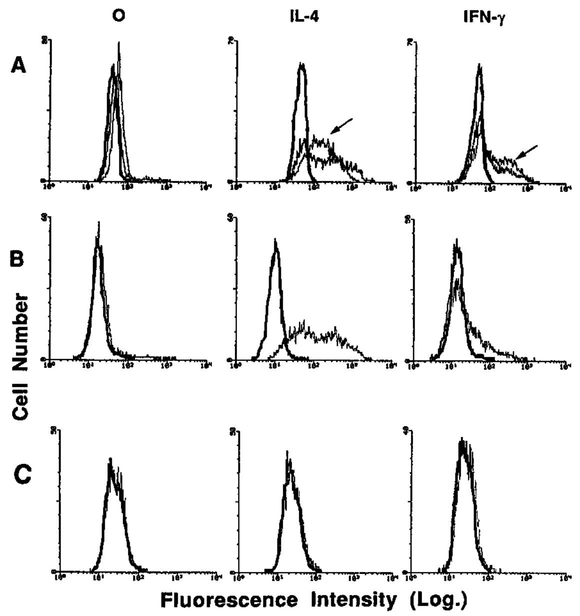

FIGURE 1 Both MHC class II molecules and invariant chain expression can be induced by IL-4 and IFN-γ in the C2.13 pro-B-cell clone. (A) Internal staining of C2.13 cells grown in medium alone (0), after 48 hr in medium supplemented with 20% of IL-4 containing supernatant or with 50 U/ml of recombinant mouse IFN-γ. Both MHC class II and Ii expression is increased internally. Profile corresponding to invariant chain is indicated by an arrow. (B) Class II expression is increased on the surface of C2.13 cells. (C) No induction of MHC class II molecules at the surface of B16.14 stromal cells by IL-4 or IFN-γ.

ments, dead cells were excluded by ethidium bromide staining, as explained in Materials and Methods.

Among three other pro-B-cell clones tested, two were inducible by both IL-4 and IFN-γ (A1.5 and F2.2), whereas D2.1 remained consistently negative (not shown). Heterogeneity in response to IL-4 has also been shown within mature splenic B cells (Noelle et al., 1986; Whitley et al., 1993). In accordance with previous reports, IFN-γ did not induce class II expression at the surface of the more differentiated 70Z/3 pre-B cells (Polla et al., 1986; and not shown). Several other compounds, such as cholera toxin or TNFα, have been shown to be able to induce class II expression on mature B cells or macrophages, respectively (Anastassiou et al., 1990; Zimmer and Jones, 1990). These compounds were not inducers for our pro-B cells (not shown).

The fibroblastoid B16.14 cell line has been derived from IL-7 transgenic bone-marrow cells infected with a recombinant retrovirus encoding a thermosensitive form of the SV40 T antigen (Fisher et al., 1993). When B16.14 cells are grown at the permissive temperature of 33°C in the presence of IFN-γ, they can be induced to express class II molecules (not shown). In theory, IFN-γ should have no effect on stromal cells that have been treated with mitomycin and are kept at 37°C, the nonpermissive temperature. However to exclude any possibility that in the presence of IFN-γ, some class II molecules could still be synthesized by B16.14 cells shed from the surface and then picked up by surrounding pro-B cells, we also performed class II staining on B16.14 stromal cells. Figure 1C shows that in these conditions, no expression could be detected at the surface of B16.14 cells, neither with IL-4 nor with IFN-γ.

We studied the kinetics of induction of class II molecules in the C2.13 cells. After 24 h of culture, in the presence of either IL-4 or IFN-γ, only the Ii chain was significantly induced. No significant levels of internal or surface class II molecules could be detected (Fig. 2; and not shown). After 48 hr of treatment, the level of Ii chain was slightly increased compared with 24 hr. At this time, significant amounts of class II molecules were expressed, resulting in cell-surface expression comparable to that shown in Fig. 1. Therefore, in C2.13 pro-B cells, the kinetics of induction of class II molecules seems to be equivalent in the presence of IL-4 or IFN-γ.

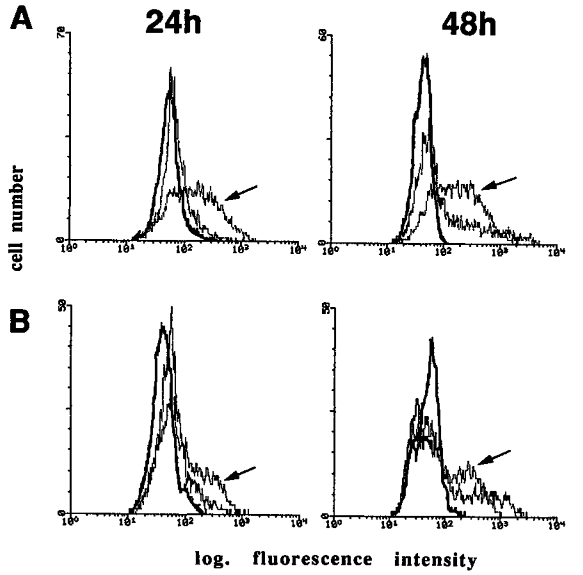

FIGURE 2 Kinetics of induction of class II molecules and invariant chain in C2.13 cells after 24 or 48 hr of treatment with (A) IL-4 or with (B) IFN-γ. C2.13 cells were internally stained for class II and Ii chain (indicated by an arrow) as explained in Materials and Methods.

The cytokine-mediated induction of class II genes has been shown to be regulated at the transcriptional level (reviewed in Ting and Baldwin, 1993). We therefore quantified the amount of Aβ mRNA in C2.13 pro-B cells following induction by cytokines. This was performed by semiquantitative RT-PCR, according to Mertsching and colleagues (1995). Control experiments were performed in which RNA preparations from noninduced IL-4 or IFN—treated cells were normalized such that the level of HGPRT transcripts, in the linear part of the PCR amplification curve, were identical, and then, RT-PCR for class II Aβ transcripts were performed (Fig. 3). Results of scanning densitometry of Aβ transcripts from the different samples showed that in two independent experiments, there was an increase of about 25-fold in the level of Aβ transcripts in IL-4-treated cells, whereas the increase by IFN-γ was only about threefold. Whether these increases in class II transcripts result from mRNA stabilization or changes in gene transcription are not known.

In mature peripheral resting B cells, IL-4 and IFN-γ have been shown to have antagonistic effects on the in-

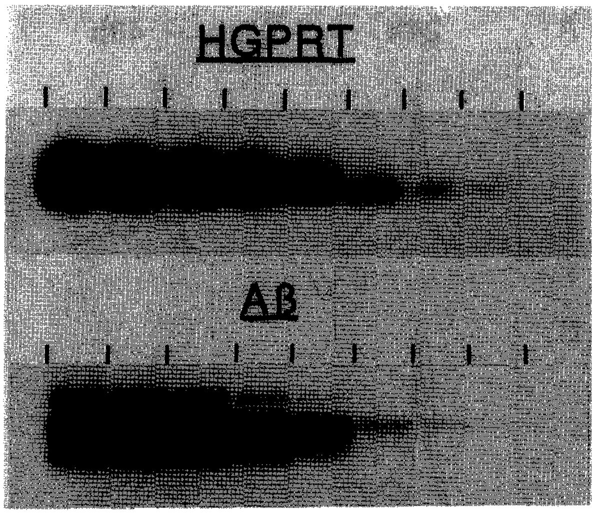

FIGURE 3 Semiquantitative RT-PCR analysis of Aβ transcripts in C2.13 cells. Shown are Southern blots of RT-PCR reactions, probed with the corresponding labeled internal oligonucleotide probes, for HGPRT (above) and Aβ (below) transcripts. The number of PCR cycles are, from left to right, 20, 22, 24, 26, 28, 30, 32, 34, and 36.

duction of class II expression: IFN-γ suppresses IL-4-induced class II expression (Mond et al., 1986). To analyze whether this was also true for pro-B cells, we have grown C2.13 cells in the presence of a low concentration of IL-4, in the absence or presence of IFN-γ. Results shown in Fig. 4 indicate that there was an additive effect on class II expression by the two cytokines.

### CONCLUDING REMARKS

In this study, we demonstrate for the first time that in pro-B cells, the expression of MHC class II molecules

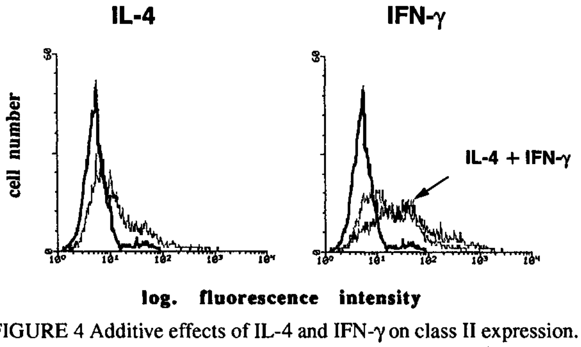

FIGURE 4 Additive effects of IL-4 and IFN-γ on class II expression. C2.13 cells were surface stained with class II mAb after 48 hr of culture in the presence of 5\- IL-4 containing supernatant, 50 U/ml of recombinant mouse IFN-γ, or both IL-4 and IFN-γ.

can be induced by both IL-4 and IFN-γ. Normally, these cytokines are the major inducers of class II in B cells and macrophages, respectively. This result suggests that the regulation of class II and Ii chain genes is developmentally regulated. That class II and Ii genes are induced by both IL-4 and IFN-γ in C2.13 pro-B cells reinforces the argument that such cells retain characteristics shared by cells of the B lymphoid and monocytic/macrophage lineages. The phenotype of C2.13 cells is clearly that of a pro-B cells, they have their IgH locus in germline configuration, they do not express monocyte/macrophage markers such as Mac-1 and F4/80, and they have a typical lymphoid morphology with a small cytoplasm (Fisher et al., 1995). At this stage, the commitment to the B cell is still reversible, because reversion to a macrophage phenotype may even be obtained with the more differentiated 70Z/3 pre-B-cell line (Tanaka et al., 1994). However, accessibility of the MHC class II promoter to IFN-γ mediated signals is lost after the pro-B-cell stage, whereas inducibility by IL-4 continues until the mature B-cell stage (Noelle et al., 1986; Polla et al., 1986; and this study).

It has been demonstrated that the activity of IFN-γ on class II and Ii genes is exerted through the same conserved S, X1, and Y DNA elements (reviewed in Brown et al., 1993; Ting and Baldwin, 1993), whereas IL-4 mediates its activity through a distal upstream region of the class II promoter (Gravallese et al., 1991). IFN—mediated induction of MHC class II expression is regulated by the transactivator CIITA (Steimle et al., 1994). Work is in progress to determine if both IL-4 and IFN-γ operate via similar or distinct intracellular signals in C2.13 cells.

### MATERIALS AND METHODS

### Cell Lines

The C2.13, D2.1, F2.2, and A1.5 pro-B-cell clones have been derived from different tumors arising in H-2d or H-2b IL-7 transgenic mice, and have been described elsewhere (Fisher et al., 1995). The cells have been cloned three times on IL-7-producing bone-marrow stromal cells B16.14. This latter cell line has been established after immortalization of an adherent primary bone-marrow stromal-cell culture from transgenic mice, as already described (Fisher et al., 1993).

### Cell Culture

C2.13 cells were grown in 7% CO2 at 37°C in DMEM (Gibco BRL, Grand Island, NY) supplemented as previously described on a monolayer of mitomycin C-treated (Sigma Chemicals, St. Louis) B16.14 stromal cells (Lombard-Platet et al., 1995). IL-4 containing supernatant was harvested from X63 myeloma cells transfected with an expression vector encoding mouse IL-4 cDNA (Karasuyama and Melchers, 1988). Recombinant mouse IFN-γ was purchased from Genzyme (Cambridge, MA).

### Flow Cytometry

To analyze the cell-surface expression of MHC class II molecules, rat culture supernatant containing the monoclonal antibody IgG2b M5/114.15.2 (ATCC TIB120) was used. This mAb detects I-Ab, I-Ad, I-Aq, and I-Ek molecules (Bhattacharya et al., 1981). Negative control was made by incubation of the rat IgG2b H35.17 CD8-specific antibody (Pierres et al., 1982). After staining, cells were fixed in 1% paraformaldehyde. For cell-surface staining, ethidium bromide (1 μg/ml) was added 2 min before analysis in order to stain and exclude dead cells in the samples. Intracellular staining was performed as described (Lombard-Platet et al., 1995), with the rat In 1.1 antibody for detection of the invariant chain (Koch and Hammerling, 1982). Flow cytometric analyses were performed on a FACScan (Becton Dickinson, Mountain View, CA).

### RNA and cDNA Preparation

After treatment with IL-4 or IFN-γ, C2.13 pro-B cells were harvested and purified from residual B16.14 stromal cells after centrifugation on a Lympholyte-M density gradient (Cedarlane, Hornby, Canada). Total RNA was isolated by the guanidinium isothiocyanate method (Maniatis et al., 1982). cDNA was synthesized as described with 3 μg of RNA, using oligo-dT as a primer (Mertsching et al., 1995).

### Reverse-Transcription PCR Assay

### *Hypoxanthine Guanine Phosphoribosyl Transferase (HGPRT) Transcripts*

RT-PCR was performed on cDNA with two primers located in different exons of the HGPRT gene 5`-GTAATGATCAGTCAACGGGGGAC-3` and 5`-CCAGCAAGCTTGCAACCTTAACCA-3`. The primers were used at 10 pmol/μl in a PCR reaction containing 5 μl of 10 × PCR buffer, 2.5 μl dNTPs (Mertsching et al., 1995), and 1U Taq polymerase (Perkin-Elmer Cetus, Norwalk, CT). Annealing temperature was 65°C. The RT-PCR reaction product was probed with a labeled internal oligonucleotide 5`-TGACACTGGTAAAACAATG-3`. RT-PCR band intensities were determined by scanning densitometry using a Fuji Bio Imaging analyser.

### *MHC Class II Aβ Transcripts*

The primers were located in the first and the second exons of the Aβ gene: 5`-CTGGAATTCCCTGTGCCTTAGAGATGGC-3` and 5`-AGCTCGGTCACCGCGC-3`. PCR reaction was performed as before, with 50°C annealing temperature. The RT-PCR reaction product was probed with a labeled internal nucleotide 5`-CTCGCCCAGGAACTGGTA-3`. Scanning densitometry was performed as before.

### *Acknowledgements*

The authors wish to thank Dr. Ronald Rooke for advice on the Aβ RT-PCR and E. Mertsching for help with the semiquantitative PCR. This work was supported in part by funds from the Institut National de la Santé et de la Recherche Médicale (INSERM), the Centre National de la Recherche Scientifique (CNRS), the Centre Hospitalier Universitaire Régional, and a grant from l'Association pour la Recherche sur le Cancer (ARC no. 6064).

### References

Anastassiou, E. D., Yadama, H., Francis, M. L., Mond, J. J., and Tsokos, G. C. (1990). Effect of cholera toxin on human B cells. **145**: 2375–2380.

Bhattacharya, A., Dorf, M. E., and Springer, T. A. (1981). A shared alloantigenic determinant on Ia antigens encoded by the I-A and I-E sub-regions: Evidence for I region gene duplication. *J. Immunol.* **127**: 2488–2495.

Brown, A. M., Wright, K. L., and Ting, J. P.-Y. (1993). Human major complex histocompatibility complex class II-associated invariant chain promoter. *J. Biol. Chem.* **268**: 26328–26333.

Cumano, A., Paige, C. J., Iscove, N. N., and Brady, G. (1992). Bipotential precursors of B cells and macrophages in murine fetal liver. *Nature* **356**: 612–615.

Fisher, A. G., Burdet, C., Bunce, C., Merkenschlager, M., and Ceredig, R. (1995). Lymphoproliferative disorders in IL-7 transgenic mice: Expansion of immature B cells which retain macrophage potential. *Int. Immunol.* **7**: 415–423.

Fisher, A. G., Burdet, C., Lemeur, M., Haasner, D., Gerber, P., and Ceredig, R. (1993). Lymphoproliferative disorders in an IL-7 transgenic mouse line. *Leukemia* **(Sup. 2)**: S66–S68.

Glimcher, L. H., and Kara, C. J. (1992). Sequences and factors: A guide to MHC class II-transcription. *Annu. Rev. Immunol.* **10**: 13–49.

Gravallese, E. M., Darling, J. M., Glimcher, L. H., and Boothby, M. (1991). Role of lipopolysaccharide and IL-4 in control of transcription of the class II Aα gene. *J. Immunol.* **147**: 2377–2383.

Grawunder, U., Melchers, F., and Rolink, A. (1993). Interferon-γ arrests proliferation and causes apoptosis in stromal cell/interleukin-7-dependent normal murine pre-B cell lines and clones in vitro, but does not induce differentiation to surface immunoglobin-positive B cells. *Eur. J. Immunol.* **23**: 544–551.

Hardy, R. R., Carmack, C. E., Li, Y. S., and Hayakawa, K. (1994). Distinctive developmental origins and specificities of murine CD5+ B cells. *Immunol. Rev.* **137**: 91–118.

Hayakawa, K., Tarlinton, D., and Hardy, R. R. (1994). Absence of MHC class II expression distinguishes fetal from adult B lymphoiesis in mice. *J. Immunol.* **152**: 4801–4807.

Karasuyama, H., and Melchers, F. (1988). Establishment of mouse cell lines which constitutively secrete large quantities of interleukin 2, 3, 4, or 5. *Eur. J. Immunol.* **18**: 97–104.

Koch, N., and Hammerling, G. J. (1982). Ia invariant chain detected on lymphocyte surfaces by monoclonal antibody. *Nature* **299**: 644–645.

Lombard-Platet, S., Fisher, A. G., Meyer, V., and Ceredig, R. (1995). Expression of functional MHC class II molecules by a mouse pro-B cell clone. *Dev. Immunol.* **4**: 85–92.

Mertsching, E., Burdet, C., and Ceredig, R. (1995). IL-7 transgenic mice: Analysis of the role of IL-7 in the differentiation of thymocytes *In vivo* and *in vitro*. *Int. Immunol.* **7**: 401–414.

Miki, N., Hatano, M., Wakita, K., Imoto, S., Nishikawa, S. I., and Tokuhisa, T. (1992). Role of I-A molecules in early stages of B cell maturation. *J. Immunol.* **149**: 801–807.

Momburg, F., Koch, N., Möller, P., Moldenhauer, G., Butcher, G. W., and Hammerling, G. J. (1986). Differential expression of Ia and Ia-associated invariant chain in mouse tissues after in vivo treatment with IFN-γ. *J. Immunol.* **136**: 940–948.

Mond, J. J., Carman, J., Sarma, C., Ohara, J., and Finkelman, F. D. (1986). Interferon-γ suppresses B cell stimulation factor (BSF-1) induction of class II MHC determinants on B cells. *J. Immunol.* **137**: 3534–3537.

Noelle, R. J., Kuziel, W. A., Maliszewski, C. R., McAdams, E., Vitetta, E. S., and Tucker, P. W. (1986). Regulation of the expression of multiple class II genes in murine B cells by B cell stimulatory factor-1 (BSF-1). *J. Immunol.* **137**: 1718–1723.

Pierres, M., Goridis, C., and Golstein, P. (1982). Inhibition of murine T-cell mediated cytolysis and T cell proliferation by a rat monoclonal antibody immunoprecipitating two lymphoid cell surface polypeptides of 94,000 and 180,000 molecular weight. *Eur. J. Immunol.* **12**: 60–69.

Polla, B. S., Poljak, A., Ohara, J., Paul, W. E., and Glimcher, L. H. (1986). Regulation of class II gene expression: Analysis in B cell stimulatory factor 1-inducible murine pre-B cell lines. *J. Immunol.* **137**: 3332–3337.

Rolink, A., Karasuyama, H., Haasner, D., Grawunder, U., Martensson, I. L., Kudo, A., and Melchers, F. (1994). Two pathways of B-lymphocyte development in mouse bone marrow and the roles of surrogate L chain in this development. *Immunol. Rev.* **137**: 1: 85–202.

Steimle, V., Siegrist, C.-A., Mottet, A., Lisowska-Gropierre, B., and Mach, B. (1994). Regulation of MHC class II expression by interferon-γ mediated by the transactivator CIITA. Science **265**: 106–109.

Tanaka, T., Wu, G. E., and Paige, C. J. (1994). Characterization of the B cell-macrophage lineage transition in 70Z/3 cells. *Eur. J. Immunol.* **24**: 1544–1548.

Tarlinton, D. (1993). Direct demonstration of MHC class II surface expression on murine pre-B cells. *Int. Immunol.* **5**: 1629–1635.

Ting, J. P.-Y., and Baldwin, A. S. (1993). Regulation of MHC gene expression. *Curr. Opin. Immunol.* **5**: 8–16.

Viville, S., Neefjes, J., Lotteau, V., Dierich, A., Lemeur, M., Ploegh, H., Benoist, C., and Mathis, D. (1993). Mice lacking the MHC class II–associated invariant chain. *Cell.* **72**: 635–648.

Whitley, M. Z., Cheng, H.-L., Tomasi, T. B., and Boothby, M. (1993). Distinct IL-4 response mechanisms of the MHC gene Aα in different mouse B cell lines. *Molec. Immunol.* **30**: 821–832.

Zhu, L., and Jones, P. P. (1990). Transcriptional control of the invariant chain gene involves promoter and enhancer elements common to and distinct from major histocompatibility complex class II genes. *Mol. Cell. Biol.* **10**: 3906–3916.

Zimmer, T., and Jones, P. P. (1990). Combined effects of tumor necrosis factor-α, prostaglandin E2, and corticosterone on induced Ia expression on murine macrophages. *J. Immunol.* **145**: 1167–1175.

